# Primary urothelial carcinoma of an ileal conduit; six decades after childhood bladder exstrophy surgery: a rare and late complication

**DOI:** 10.1186/s12957-025-03798-y

**Published:** 2025-05-31

**Authors:** Areeba Ahmed, Sameen Nasir, Imran Khan Jalbani, Amna Qadri

**Affiliations:** 1 Department of Surgery, Section of Urology, Aga Khan University, Karachi, Pakistan; 2 Medical College, Aga Khan University, Karachi, Pakistan; 3 Department of Pathology, Aga Khan University, Karachi, Pakistan

**Keywords:** Urothelial carcinoma, Bladder exstrophy, Ileal conduit, Chemotherapy, Cystoscopy

## Abstract

**Background:**

Bladder exstrophy is a rare congenital anomaly that requires surgical reconstruction or urinary diversion early in life. While adenocarcinoma is the most commonly associated malignancy, primary urothelial carcinoma arising within an ileal conduit without any evidence of disease in the entire urinary tract is exceedingly rare and has never been reported before.

**Case presentation:**

We report a case of a 64-year-old male with a history of bladder exstrophy managed with an ileal conduit in early childhood. He presented with intermittent bleeding from his urinary stoma, and subsequent evaluation revealed a high-grade invasive urothelial carcinoma arising within the ileal conduit, without involvement of the ureteric orifices or native urinary tract. Metastatic spread to the regional lymph nodes and liver underscored the aggressive disease course. Despite prompt initiation of chemotherapy and later immunotherapy, the disease progressed rapidly, leading to severe complications, including bilateral hydronephrosis requiring percutaneous nephrostomy. The patient was ultimately transitioned to palliative care.

**Conclusion:**

Primary urothelial carcinoma in an ileal conduit of bladder exstrophy patient is a rare condition. The latency period for the onset of this aggressive cancer in urinary diversions can be long but mainly occurs before the age of 65. This reinforces the need for long-term follow-up of patients with urinary diversions, even in the absence of symptoms. We advocate for routine screening of these patients, initiating before the age of 30 as previously recommended for bladder exstrophy patients.

## Introduction

Bladder exstrophy (BE) is a rare congenital anomaly in which the bladder is exposed outside the abdominal wall due to incomplete closure during fetal development. It is part of a broader spectrum known as the bladder exstrophy-epispadias complex (BEEC), which includes additional anomalies such as epispadias and cloacal exstrophy, affecting the urinary tract, genitalia, bony pelvis, and other structures. The diagnosis of BE is primarily clinical, with further investigations typically unnecessary [1]. The modern approach to management involves primary repair of the anomaly. However, in cases where the bladder is too small and fibrotic, urinary diversion with cystectomy becomes the preferred treatment [2, 3]. Patients with bladder exstrophy remain at an increased risk of urological complications and malignancies, even decades after their initial surgery. The lifetime risk of developing neoplasia in adults born with exstrophy is reported to be as high as 17.5%. Additionally, despite undergoing bladder closure or diversion surgery early in life, these patients have a 700-fold higher incidence of bladder cancer compared to the age-matched general population. This was first highlighted by Smeulders and Woodhouse in 2001, demonstrating that early cystectomy does not confer protection [4]. Adenocarcinoma is the most frequently reported malignancy associated with bladder exstrophy, attributed to mucosal metaplasia of intestinal epithelial elements. While adenocarcinoma is the predominant histological type, cases of squamous cell carcinoma and, rarely, transitional cell carcinoma in untreated bladder exstrophy, have also been documented [5–8] To date, there have been no reported cases of primary urothelial carcinoma arising within an ileal conduit decades after initial surgical repair, without a prior history of malignancy. Here, we present the first reported case of an elderly male with a history of urinary diversion (ileal conduit) for bladder exstrophy in childhood, who was subsequently diagnosed with primary urothelial carcinoma of the ileal conduit with metastases. Notably, no evidence of urothelial cancer was found elsewhere in the urinary tract.

### Case presentation

A 64-year-old male with a history of type 2 diabetes, chronic kidney disease (CKD), hypercholesterolemia, and hypothyroidism presented to the outpatient department with a two-month history of intermittent bleeding from his urinary stoma. He was born with bladder exstrophy and underwent ileal loop urinary diversion at the age of two. Following surgery, he adapted well, learned to empty his bladder through the conduit, and remained free of serious complications until the age of 64.

On general physical examination, the patient appeared mildly pale but was otherwise in good overall health. Abdominal examination revealed a urinary stoma in the right lower quadrant.

Laboratory investigations showed a normal complete blood count, glycated hemoglobin, and serum creatinine. Ultrasound imaging revealed bilateral renal cortical thinning, irregular contours, and fullness of the collecting system and ureters, consistent with chronic pyelonephritis. No significant abnormalities were detected within the ileal conduit at that time.

**Fig. 1:**
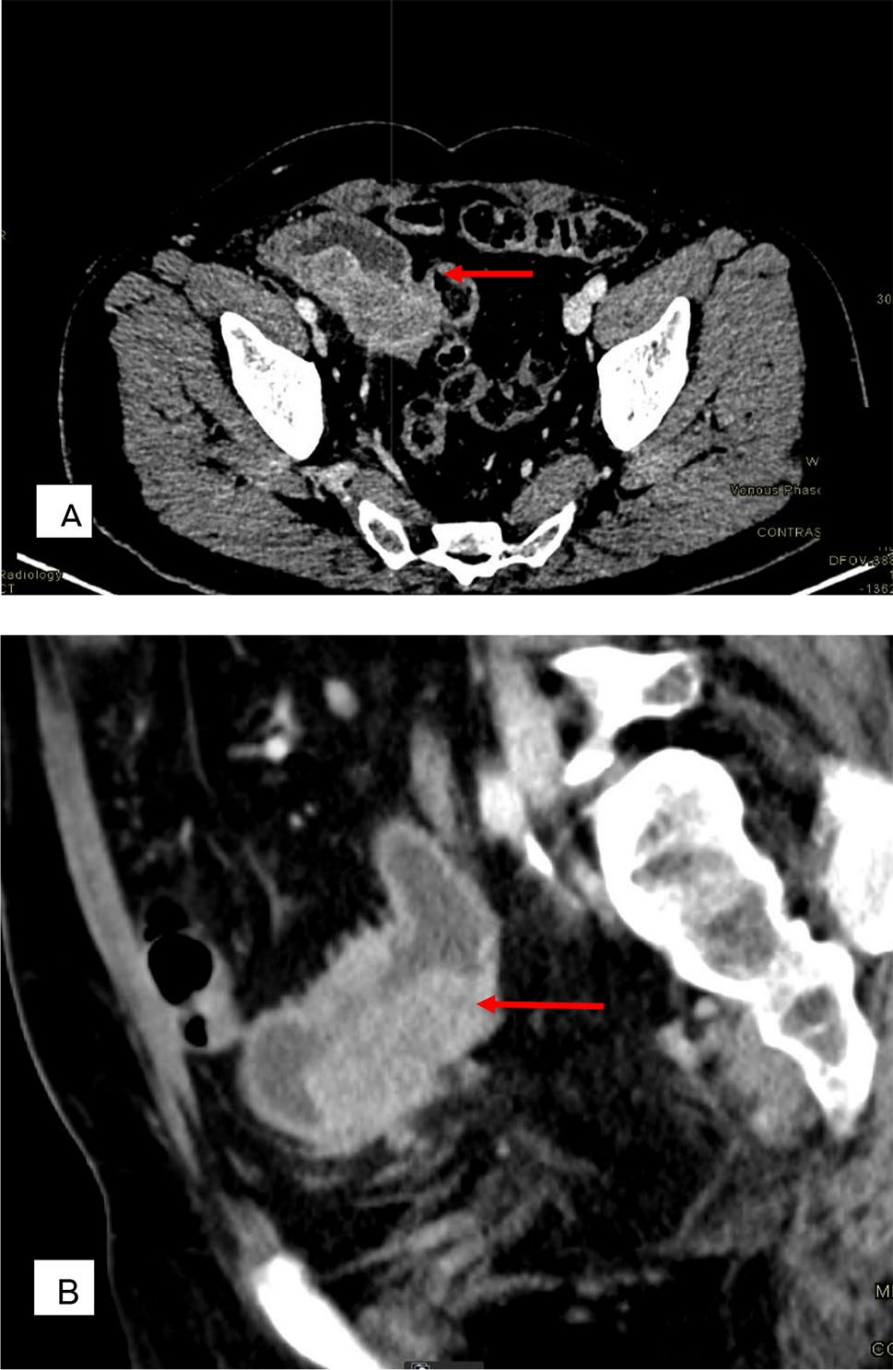
Primary urothelial carcinoma of the ileal conduit; Six decades after childhood bladder exstrophy surgery (**A**) Axial view demonstrating ileal conduit mass (**B**) Coronal view

The patient was lost to follow-up for two months. Upon returning to the clinic, persistent hematuria prompted further evaluation with a contrast-enhanced computed tomography (CECT) scan of the abdomen. The scan revealed a large, enhancing soft tissue mass (71 × 28 mm) within the ileal conduit, without involvement of the uretero-ileal anastomosis. Additionally, enlarged regional lymph nodes were identified, with the largest measuring 15 mm. At this stage, no evidence of distant metastasis to the lungs, bones, or liver was detected. (Fig. 1)

Given these findings, a biopsy was scheduled for the following week, and a flexible cystoscopy of the ileal conduit was performed under local anesthesia. During the procedure, a 6 × 6 cm polypoidal growth was identified in the distal part of the conduit, located 4 cm distal to the ureteric orifices. An endoscopic biopsy was obtained, confirming that the tumor did not involve the ureteric orifices. The patient tolerated the procedure well and remained hemodynamically stable.

**Fig. 2:**
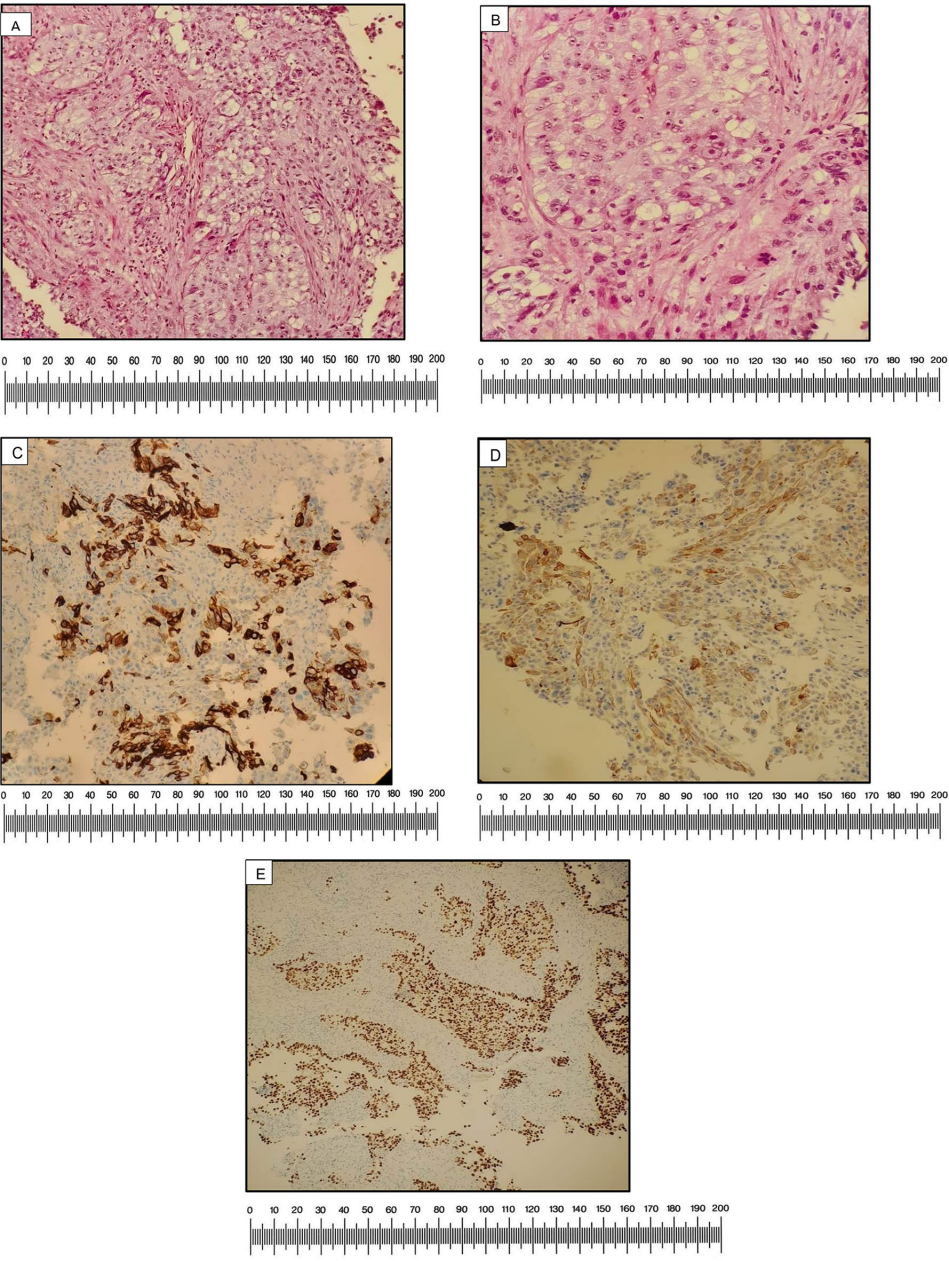
(**A**) Low power (10X): Tissue fragments involved by a neoplastic lesion showing nests and clusters of polygonal atypical epithelial cells in the desmoplastic stroma (**B**) High Power (40X): Individual cells are polygonal and markedly pleomorphic showing vesicular chromatin and prominent nucleoli. The cytoplasm is abundant and pale (**C**) CK7: Patchy positive in tumor cells (**D**) CK 20: Patchy positive in tumor cells (**E**) P63: Positive in tumor cells

Histopathological analysis of the biopsy specimen confirmed a high-grade invasive urothelial carcinoma. Immunohistochemical staining demonstrated patchy positivity for cytokeratin 7 (CK7) in tumor cells, while cytokeratin 20 (CK20) exhibited patchy and dim positivity, a profile consistent with urothelial origin. This was further supported by diffuse positivity for tumor protein 63 (p63), confirming urothelial differentiation. Caudal-type homeobox transcription factor 2 (CDX2), a marker of gastrointestinal origin, was negative, reinforcing the diagnosis of primary urothelial carcinoma. (Fig. 2)

Following the biopsy results, a Fluorodeoxyglucose Positron Emission Tomography (FDG PET) /computed tomography scan was performed to assess the extent of the disease. The scan identified a hypermetabolic soft tissue mass within the ileal conduit in the right lower quadrant, corresponding to the previously biopsied lesion. The mass measured 62 × 34 mm and had a maximum Standardized Uptake Value (SUV) of 32. Additionally, metastatic hypermetabolic lymph nodes were detected in the right pelvis (19 × 24 mm, SUV 21), along with a solitary liver metastasis in segment III (24 × 20 mm, SUV 15.5). No hypermetabolic lymph nodes were observed in the para-aortic or inguinal regions.

**Fig. 3:**
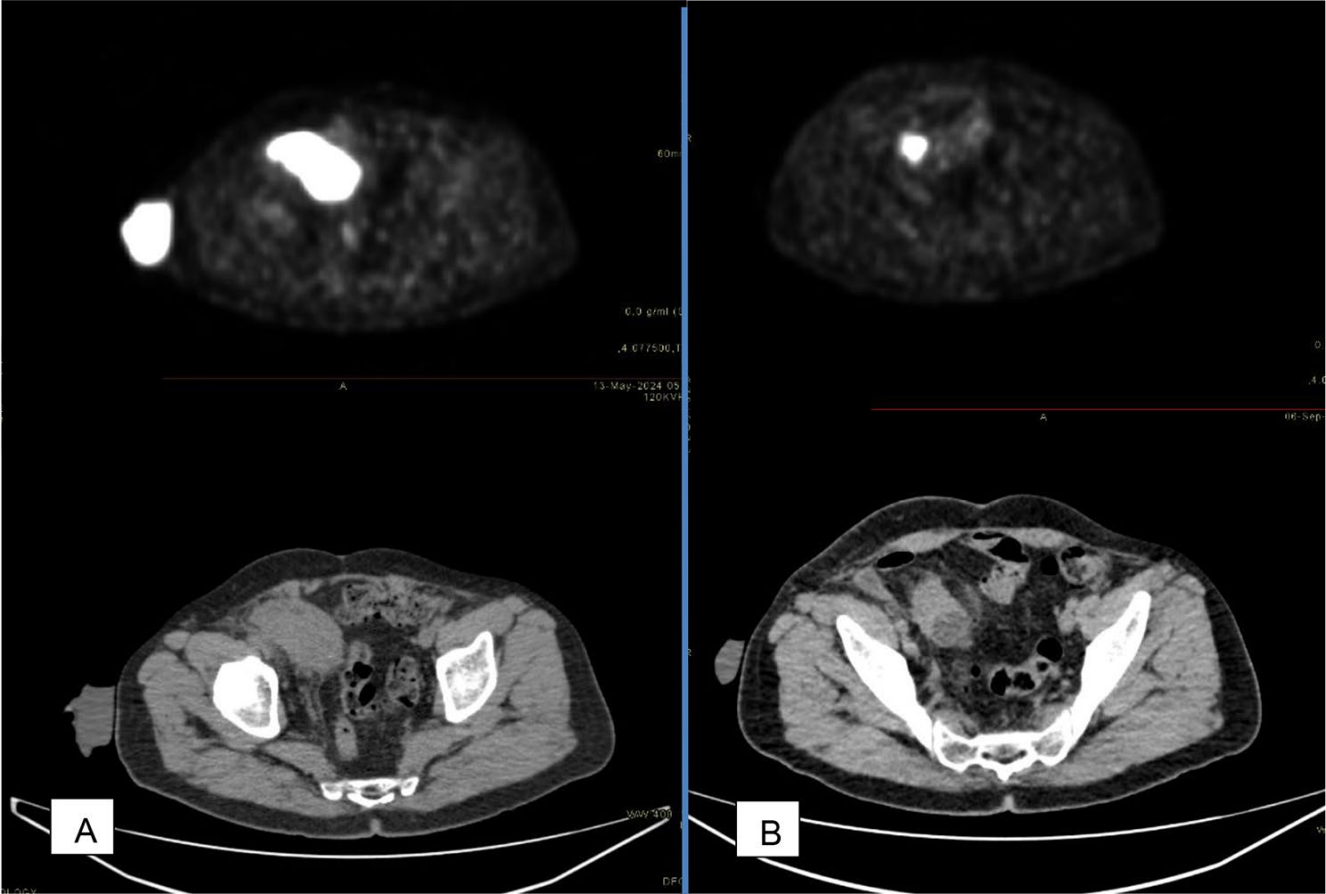
(**A**) FDG PET/CT scan before initiation of chemotherapy (**B**) Post-chemotherapy scan

Given the diagnosis of high-grade invasive urothelial carcinoma with metastatic disease involving the liver, the patient was referred to the medical oncology team and initiated on a chemotherapy regimen of carboplatin and gemcitabine the following month. An FDG PET/CT scan performed three months after the initial imaging showed a reduction in tumor size following six cycles of chemotherapy (Fig. 3). However, the scan also revealed bilateral hydronephrosis secondary to tumor obstruction within the ileal conduit. With progressively worsening renal function, the patient was referred back to the urology department for management of obstructive uropathy, and bilateral percutaneous nephrostomy tubes were placed. During his hospital stay, his serum creatinine levels showed improvement. The patient and his family opted against stoma refashioning.

On repeat FDG PET/CT scan eight months after the initial histopathological diagnosis, disease progression was observed. Consequently, the oncology team switched treatment to immunotherapy with pembrolizumab. However, after two doses over one month, the patient continued to exhibit clinical disease progression, leading to the discontinuation of immunotherapy. As his general health declined, he was referred to palliative care. He is currently being maintained on bilateral nephrostomy.

## Discussion

As the literature reports, carcinogenesis is a recognized phenomenon in bladder exstrophy patients [4, 9, 10]. It was previously thought that carcinoma occurred only in untreated cases of bladder exstrophy. While adenocarcinoma is the most common malignancy associated with this congenital condition, a few cases of urothelial carcinoma have also been reported in untreated bladder exstrophy [8, 11, 12]. Later, it was interpreted that the risk of carcinoma remains elevated even after surgical repair of the anomaly [13]. 

Literature suggests that patients who undergo any form of urinary diversion have an increased cancer risk. Ko Joan et al. reported a case of a bladder exstrophy patient who developed adenocarcinoma within the ileal conduit, involving the left ureter. It is postulated that chronic exposure of urinary conduit to urine induces histologic changes of chronic inflammation, which may play a role in carcinogenesis. Inflammatory cell-derived substances can promote dysplasia and eventual malignancy [13]. Additionally, the use of an ileal segment for urinary diversion may further contribute to this risk. The ileal mucosa is not naturally adapted to prolonged exposure to urine, particularly its hydrogen ion concentration (pH). Over time, this exposure may lead to metaplastic changes, such as intestinal metaplasia, thereby increasing the likelihood of malignant transformation.

In our case, urothelial carcinoma developed within the ileal conduit without any prior history of bladder malignancy, a phenomenon that has not been previously reported. The occurrence of high-grade invasive urothelial carcinoma in the ileal conduit, with no evidence of malignancy elsewhere in the urinary tract, is particularly noteworthy. One possible explanation is the migration of the urothelial epithelium from the urinary tract into the conduit. The patient exhibited an aggressive disease course, with metastatic spread to the liver and regional lymph nodes, emphasizing the highly malignant nature of urothelial carcinoma. Despite early diagnosis and timely initiation of chemotherapy as well as immunotherapy, the disease progressed rapidly, leading to severe complications such as ileal conduit obstruction, which required additional interventions. Our case not only highlights the potential for urothelial cancer to follow a relentless trajectory, often resulting in significant morbidity and a challenging clinical outcome but also reinforces the need for long-term follow-up for patients with urinary diversions, even in the absence of symptoms.

Morikawa et al. reported a case of a bladder exstrophy patient who underwent urinary diversion and substitution cystoplasty with Mitrofanoff appendicovesicostomy in teenage and developed invasive bladder carcinoma in the de-functionalized bladder at the age of 44 [14]. The latency period for the onset of cancer in urinary diversions can be extremely long, as seen in most of the cases including ours, where the patient presented with urothelial carcinoma 60 years after his initial surgery. Arkani et al. conducted a pioneering study on bladder cancer in bladder exstrophy-epispadias complex (BEEC) patients, analyzing 12 cases from the Swedish national registry and summarizing 165 cases reported in the literature. Their findings emphasize that, despite a long latency period, bladder cancer in BEEC patients tends to present at a relatively younger age, with the majority of cases diagnosed before 65 years, highlighting the early-onset nature of malignancy in this population [15]. 

Despite being widely discussed and suggested by various authors, screening for bladder cancer in individuals with BEEC has yet to be formalized into official guidelines [16–18]. 

We have learned that early diagnosis is key to a better prognosis; therefore, early regular screening for cancer should be implemented in individuals with BEEC. A screening approach combining cystoscopy, urinary cytology, and targeted biopsies of the ileal conduit may be helpful. Based on an extensive literature review, we strongly advocate for initiating screening before the age of 30 and annually thereafter, aligning with the prior recommendations of Arkani et al. [15] Given the diagnostic challenges associated with malignancy in BEEC patients, we also emphasize the importance of maintaining a high index of suspicion for carcinoma in those presenting with hematuria, refractory lower urinary tract symptoms, or unexplained weight loss. In particular, cases of gross hematuria should never be overlooked and must be promptly evaluated.

## Conclusion

Urothelial carcinoma is a highly aggressive malignancy, particularly in patients with bladder exstrophy and early urinary diversion. We emphasize the importance of regular surveillance, including periodic endoscopic evaluation of the ileal conduit and urine cytology, to enable early detection and timely intervention. Implementing such measures could significantly improve patient outcomes, even in complex cases like ours, where multidisciplinary management involving urologists, oncologists, and interventional radiologists is essential for optimal care.

## Data Availability

No datasets were generated or analysed during the current study.

## Abbreviations

BE: Bladder exstrophy
BEEC: Bladder exstrophy–epispadias complex
CDX2: Caudal–type homeobox 2
CECT: Contrast–enhanced computed tomography
CK7: Cytokeratin 7
CK20: Cytokeratin 20
CKD: Chronic kidney disease
CT: Computed tomography
FDG: Fluorodeoxyglucose
PET: Positron emission tomography
SUV: Standardized uptake value
